# Expectations of cerebrospinal fluid shunt treatment outcome in idiopathic normal pressure hydrocephalus: a combined cross-sectional and prospective cohort study

**DOI:** 10.1016/j.bas.2026.106664

**Published:** 2026-09-05

**Authors:** Michael P. Meier, Reuben Christopher, Alexander Romagna, David B. Schul, Samira Hintze, Jens Lehmberg

**Affiliations:** a Department of Neurosurgery, München Klinik Bogenhausen, Englschalkinger Straße 77, Munich, 81925, Germany; b Department of Neurosurgery, University Hospital Salzburg, Paracelsus Medical University, Ignaz-Harrer-Straße 79, Salzburg, 5020, Austria; c Department of Neurosurgery, InnKlinikum Altötting, Vinzenz-von-Paul-Str. 10, Altötting, 84503, Germany

**Keywords:** Idiopathic normal pressure hydrocephalus (iNPH), Shunt surgery / cerebrospinal fluid shunting, Treatment expectation, Caregiver expectation, Treatment outcome perception

## Abstract

**Introduction:**

Idiopathic normal pressure hydrocephalus (iNPH) is increasingly relevant in the aging population. Despite improved diagnostic criteria, postoperative outcome remains variable and difficult to predict. Preoperative expectations may substantially influence perceived treatment success, and caregivers often play a key role in the decision-making process.

**Research question:**

This study evaluated whether physicians assess postoperative outcomes more accurately than primary caregivers.

**Material and methods:**

This single-centre study combined a prospective pre–post interventional cohort design with a cross-sectional component. Patients with iNPH undergoing first-time ventriculoperitoneal shunt surgery were included. Symptom severity was assessed preoperatively and at follow-up using standardized grading scales. Primary caregivers (PCGs), neurosurgical residents, and board-certified neurosurgeons independently predicted postoperative outcomes, which were subsequently compared with observed treatment results.

**Results:**

Forty-one patients were screened between January 2022 and June 2025. The study cohort had a mean age of 74.1 years (SD 7.3), 57% male, mean symptom duration 23.9 months (SD 14.1) and a mean Charlson comorbidity index of 4.1 (SD 1.3). At a median follow-up of 48 days (range 36–118) after ventriculoperitoneal shunt surgery, mean symptom improvement was Δ 2.2 points (95% CI 1.6–2.7; p < 0.001), with the greatest improvement observed in gait disturbance and urinary incontinence. Revision surgery was required in 17% of patients. Correlation between predicted and observed outcome was strongest among neurosurgical specialists (r = 0.79), followed by residents (r = 0.74), while PCGs demonstrated only moderate agreement (r = 0.56).

**Discussion and conclusion:**

Our study supports the efficacy of CSF shunt surgery in iNPH and demonstrates that neurosurgical expertise aligns more closely with actual postoperative outcome than the expectations of PCGs. Structured preoperative counselling may help improve expectation management and reduce discrepancies between anticipated and observed treatment benefit.

## Highlights

• CSF shunt surgery improves symptoms in idiopathic normal pressure hydrocephalus.
• Gait disturbance shows the strongest postoperative improvement in iNPH.
• Physician expectations correlate more closely with postoperative iNPH outcome.
• Caregivers tend to overestimate postoperative benefit after shunt treatment in iNPH.
• Expectation management may improve preoperative counselling in iNPH.

## 1 Introduction

Idiopathic normal pressure hydrocephalus (iNPH) is characterized by the accumulation of cerebrospinal fluid (CSF) within the cerebral ventricular system, resulting in ventricular enlargement and compression of the surrounding brain tissue. Normal pressure hydrocephalus is classified into idiopathic (primary) forms, where no underlying cause is identified, and secondary forms, that arise from conditions such as subarachnoid haemorrhage, meningitis, traumatic brain injury, or prior neurosurgical interventions. The characteristic triad of iNPH—gait disturbance, cognitive decline, and urinary incontinence—often manifests gradually, with gait abnormalities preceding cognitive or urinary symptoms (Sundström et al., 2022; Wikkelso et al., 2013). The prevalence of iNPH increases significantly with age; among individuals over the age of 65, its occurrence is estimated to be 0.5%–8.9% (Jaraj et al., 2014; Andersson et al., 2019; Nakajima et al., 2021).

Despite progressively refined diagnostic criteria over the past decades, NPH remains challenging to diagnose due to symptom overlap with neurodegenerative disorders such as Alzheimer's and Parkinson's diseases (Martín-Láez et al., 2015). Neuroimaging and invasive CSF testing are central components of diagnostic assessment. Diversion of excess CSF can significantly improve motor symptoms, although the effect on cognitive function and urinary recovery may be less pronounced (Luciano et al., 2025; Kazui et al., 2015; Pearce et al., 2024). Treatment response may decline over time, and some patients do not respond to treatment, highlighting the uncertainty of treatment outcome and the need for valid diagnostic criteria (Schniepp et al., 2017).

Patient satisfaction and therapeutic success depend largely on preoperative expectations (Soroceanu et al., 2012). Unrealistic or poorly managed expectations may reduce satisfaction, hinder rehabilitation, and lead to repeated medical consultations. Across surgical fields, patients and their relatives maintain higher expectations for surgical outcomes than their treating physicians (Lattig et al., 2013; Rönnberg et al., 2007).

Cognitive deficits in iNPH often result in patients’ relatives assuming a central role in daily care and becoming key initiators of medical treatment. Integration of caregivers into counselling and decision-making may improve expectation management and treatment satisfaction (Toma et al., 2011; Subramanian et al., 2018).

This study used a prospective, single-centre pre-post interventional cohort design with a cross-sectional component. It aimed to evaluate iNPH outcomes using established grading systems and to examine how these outcomes relate to the expectations of different raters. Patients' primary caregivers (PCGs) and medical professionals were asked to provide their outcome expectations following CSF shunt surgery, which were subsequently compared with observed postoperative results. The study was designed to test the hypothesis that physicians assess surgical outcomes in patients with iNPH more accurately than patients’ PCGs.

## 2 Methods

This prospective trial included patients with a diagnosis of iNPH scheduled for first-time CSF shunt surgery. Symptom severity was assessed at baseline and at follow-up (f/u). At baseline, anticipated postoperative outcomes were independently estimated by the patient's PCG, a neurosurgical resident, and a board-certified neurosurgeon. The expectations of the three rater groups were subsequently correlated with observed patient outcomes 6 weeks after surgery. Reporting followed the STROBE guidelines for cohort and cross-sectional studies (von Elm et al., 2007).

### 2.1 Setting

Study ethic board approval (ID 649/21 S) was received in 11/2021. Patient recruitment was completed from January, 2022, to June, 2025, with the last (second) f/u held in December, 2025. The study was registered with the German Federal Institute for Drug and Medical Devices under the study ID: DRKS000310.

### 2.2 Data collection

Primary study data were collected using standardized evaluation sheets (ES) and outcome expectation questionnaires (OEQ) (Supplementary Materials 1–3). The ES was used to document neurological status at baseline and follow-up, whereas the OEQ assessed anticipated treatment outcome. Both instruments covered the individual domains and subcategories of the iNPH grading scale (Kubo et al., 2008), supplemented by headache and dizziness, as described in other grading scales (Kiefer et al., 2003). Symptoms of the Hakim triad were rated from 0 (absent) to 4 (severe), and headache and dizziness were differentiated as absent, recurrent, or persistent (0–2), yielding a maximum total score of 16 (0 indicating no symptoms; Table 1).

**Table 1:** iNPH Symptom Score.

Score	Gait disturbance	Cognitive impairment	Urinary incontinence	Headache	Dizziness
0	Normal	Normal	Normal	No headaches	No dizziness
1	Complaints of dizziness of drift and dysbasia but not objective gait disturbance	Complaints of amnesia or inattention but no objective memory and attentional impairment	Pollakiuria or urinary urgency	Intermittent or permanent headache which does not influence the activity of daily life	Intermittent dizziness appearing under stress or spontaneous dizziness
2	Unstable but independent gait	Existence of amnesia or inattention, but no disorientation of time and place	Occasional urinary incontinence (1-3 or more times a week but less than once per day)	Permanent severe headache	Permanent dizziness
3	Walking with any support	Existence of disorientation of time and place, but conversation is possible	Continuous urinary incontinence (1 or more time per day)		
4	Walking not possible	Disorientation for the situation or meaningful conversation impossible	Bladder function is almost or completely deficient		

The patients’ demographic and clinical data were obtained from medical records and structured interviews, including symptom duration, educational level, and the relationship between patient and PCG. Conservative treatment prior to surgery was not assessed in the study. Surgical treatment variables and adverse events were prospectively recorded.

### 2.3 Participants

Patients meeting the diagnostic criteria for iNPH and scheduled for first-time CSF shunt surgery, along with their PCG, were considered eligible for the study. Given the potential cognitive impairment associated with iNPH, outcome expectations were assessed through PCGs rather than the patients themselves.

Exclusion criteria included secondary hydrocephalus (e.g., post-haemorrhagic, occlusive, congenital), prior shunt treatment, severe disability (modified Rankin Scale ≥ 5), age <18 years, pregnancy, breastfeeding, and PCGs lacking regular patient interaction. Reliable outcome assessment was considered to require knowledge of the patients’ baseline condition. Accordingly, eligible PCGs were expected to have regular face-to-face patient contact (≥1 h at least twice weekly). Legal guardians were therefore not considered eligible as PCGs.

For the assessment of anticipated treatment outcomes, each patient was randomly assigned to one neurosurgical specialist and one neurosurgical resident. Participating physicians were required to review the patients’ medical history and imaging and to perform an independent clinical assessment, including gait evaluation and cognitive testing, as well as structured anamnesis.

### 2.4 Detailed study procedure

In accordance with international guidelines, iNPH was diagnosed based on clinical symptoms, radiological criteria of hydrocephalus, and invasive CSF investigations (Nakajima et al., 2021; Relkin et al., 2005). However, invasive testing was not deemed necessary in patients presenting with the complete Hakim triad and corresponding radiological findings. Radiological evidence of hydrocephalus included an Evans ratio of > 0.3, together with at least one of the following: enlarged temporal horns, a callosal angle < 90°, transependymal CSF flow, or narrow high-convexity sulci.

**Fig. 1:**
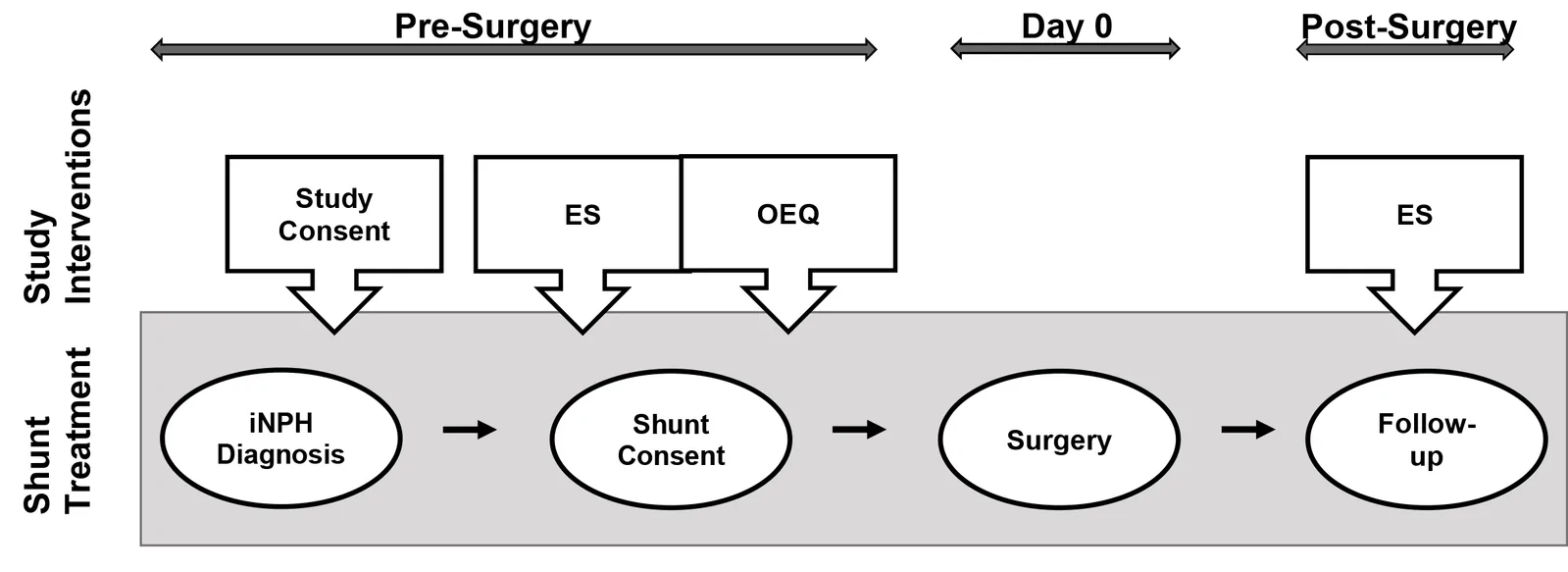
*Study timeline* showing study-related procedures (top) and treatment-related activies (bottom) relative to the CSF shunt surgery (day 0). (ES) Evaluation Sheet; (OEQ) Outcome Expecation Questionnaire.

Patients with iNPH presenting to the neurosurgical department were informed about the study. Patients and their PCG who agreed to participate provided written informed consent and were subsequently screened for eligibility. Patients consented to the pre-post interventional component, whereas PCGs consented to participation in the cross-sectional assessment. Baseline data were collected using the ES prior to initiation of the surgical approval process for CSF shunt treatment (Fig. 1). Patients and PCGs received standardized counselling regarding disease characteristics, treatment options, surgical risks, and postoperative management. Subsequently, the OEQ was completed by the patient's PCG. Thereafter, for each patient, one resident and one neurosurgical specialist, both randomly selected from the local neurosurgical department, independently provided their outcome expectations using the OEQ. None of the raters had access to the completed OEQs of the other raters. All raters received a brief standardized explanation of the questionnaire, specifying that expected outcomes 6 weeks following shunt surgery were to be assessed.

Patients and their families were encouraged to participate in postoperative rehabilitation. The primary f/u visit was scheduled 42 days after surgery, at which time a second ES was completed. Patients were further encouraged to attend an additional (second) f/u visit to assess the stability of treatment success.

### 2.5 Statistical analyses

A sample size calculation was performed. For the paired Student's t-test, a sample size of n = 34 was required (α = 0.05, power = 80%, standard deviation = 2, mean difference = 1), while for Pearson's correlation analyses, a sample size of n = 32 was calculated (α = 0.05, power = 85%, expected correlation coefficient r = 0.5).

Continuous variables are presented as mean (SD) or median (range), as appropriate, and categorical variables as absolute frequencies and percentages. Pre–post differences were analysed using paired Student's t-tests or Wilcoxon signed-rank tests, depending on data distribution, and are reported with corresponding 95% confidence intervals (95% CI). Pearson's correlation coefficient (r) was applied to evaluate linear associations between participants' expectations and observed outcomes.

All tests were two-tailed, with a significance level set at α = 0.05. Potential confounders included age, gender, educational level (≤10 vs > 10 years of schooling), overall health status (Charlson comorbidity index), duration of hydrocephalus-related symptoms, and extent of diagnostic work-up (with or without invasive investigations); these variables were examined in a series of multiple linear regression models. To account for ratings within patients, linear mixed-effects models were additionally applied using the same covariates, with patient ID included as a random intercept.

All statistical analyses were conducted using IBM SPSS Statistics (Version 27).

## 3 Results

### 3.1 Baseline characteristics

**Fig. 2:**
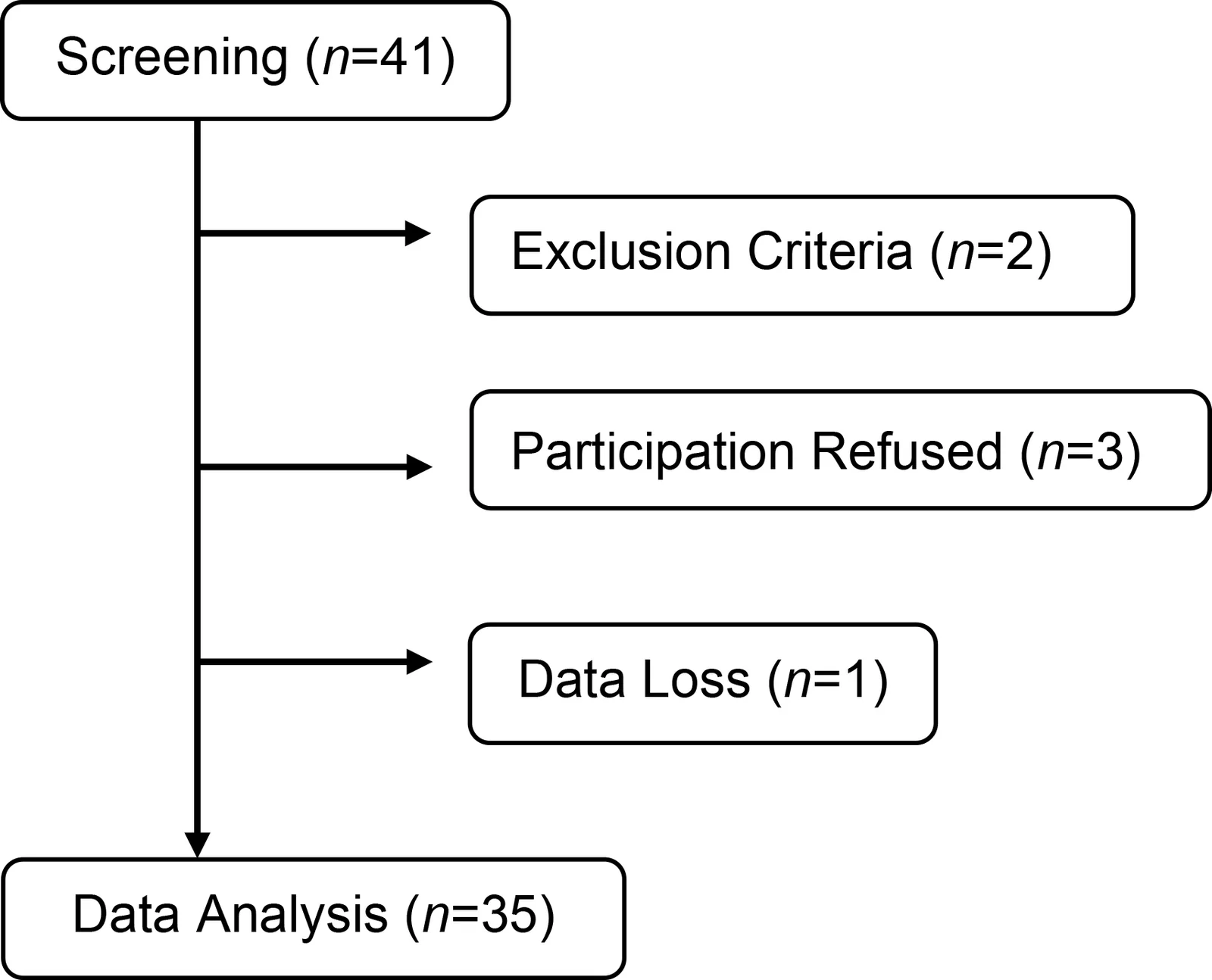
Flow diagram of study participants.

A total of 41 patients were screened for study eligibility between January 2022 and June 2025. Three patients declined participation, two patients had no eligible PCG other than a legal representative without frequent in-person contact, and one case was excluded due to data loss. No loss of follow-up occurred, resulting in a final analysis of 35 patient data sets (Fig. 2). The mean age of the iNPH patients was 74.1 years (SD 7.3). All patients suffered from comorbidities with the majority (86%) been ranked a Charlson Comorbidity Index (CCI) of ≥ 3 (mean 4.1). In 77% of cases, iNPH symptoms preceded the final diagnosis for more than 12 months (Table 2). All patients presented with gait disturbance, cognitive impairment and/or urinary incontinence and met radiological criteria for hydrocephalus as described above. Invasive CSF investigation to further support the diagnosis was performed in 71% of patients (n = 25) using a high-volume lumbar tap test; all patients reported symptom improvement following the procedure. For each included patient (n = 35), one resident, one neurosurgical specialist and one PCG independently completed an OEQ. Among the physician raters, 16 of the 35 neurosurgical specialists had more than 10 years of professional experience, while the resident raters had a mean duration of neurosurgical training of 2.9 years (SD 1.3).

**Table 2:** Baseline Characteristics, values are mean (SD) and n (%).

Patient data	*n = *	(%)
**Age** 74.1 (7.3)		
< 66 Years	4	11
≥ 66 Years	31	89
**Gender**		
Female	15	43
Male	20	57
**Formal education**		
≤10 Years	14	40
>10 Years	21	60
**CCI** 4.1 (1.3)		
CCI < 3	5	14
CCI ≥ 3	30	86
**Duration of Symptoms** 23.9 (14.1)		
< 12 Months	8	23
≥ 12 Months	27	77
**Primary caregiver**		
Spouse	18	51
Children	13	37
Relatives	4	11

### 3.2 Surgical outcome data

In all study patients a ventriculoperitoneal (VP) shunt (Codman Certas® Plus with unitized Bactiseal) was implanted. The valve opening pressure was set at a default level of 4 in all treatments, corresponding to a pressure of 110 mm H_2_0 (±25 mm H_2_0). Adverse events requiring surgical revision occurred in six patients, including shunt dislocation (n = 3), impaired wound healing, subdural hematoma and an intracerebral haemorrhage (each n = 1). Although not considered as adverse event, postoperative valve adjustments in the outpatient setting were necessary in five cases, with suspected over-drainage in four.

### 3.3 Pre-post intervention study

At baseline, mean scores for the individual ES categories were as follows: gait disturbance 2.6 (SD 0.7), cognitive impairment 2.0 (SD 0.9), urinary incontinence 2.4 (SD 1.2), headaches for 0.2 (SD 0.4) and dizziness 0.5 (SD 0.7) (Table 3). At a median follow-up of 48 days (range 36-118) after shunt implantation, all patients reported subjective improvement. A clinically relevant improvement of ≥ 1 points in the ES was observed in 83% of the study patients. The mean ES score decreased from 7.7 (SD 2.3) at baseline to 5.5 (SD 2.5) at f/u, corresponding to a mean reduction of 2.2 points (95% CI -2.7 to −1.6; p < 0.001).

**Table 3:** Outcome data, iNPH symptom status as assessed by ES, expectations as assessed by OEQ, data are mean and (SD), differences are mean and (95% CI).

	Patients (n)	iNPH Symptom Status				Expectations		
		Basline score	Follow-up score	Difference	p value	PCG	Residents	Neurosurgeons
**Gait**	35	2.6 (0.7)	1.7 (1.0)	−0.9 (−1.1 to −0.7)	<0.001	0.9 (0.9)	1.5 (0.9)	1.5 (0.7)
**Cognition**	35	2.0 (0.9)	1.7 (0.9)	−0.3 (−0.5 to 0.0)	0.03	1.0 (0.7)	1.3 (0.7)	1.3 (0.6)
**Urinary**	35	2.4 (1.2)	1.6 (1.3)	−0.8 (−1.0 to −0.6)	<0.001	0.9 (0.8)	1.6 (1.0)	1.4 (1.1)
**Headaches**	35	0.2 (0.4)	0.3 (0.6)	0.1 (−0.1 to 0.3)	>0.05	0.1 (0.2)	0.0 (0.2)	0.1 (0.3)
**Dizziness**	35	0.5 (0.7)	0.3 (0.5)	−0.3 (−0.5 to 0.0)	0.02	0.1 (0.3)	0.1 (0.3)	0.2 (0.4)
**Total**	35	7.7 (2.3)	5.5 (2.5)	−2.2 (−2.7 to −1.6)	<0.001	3.0 (2.1)	4.5 (1.8)	4.6 (2.0)

**Fig. 3:**
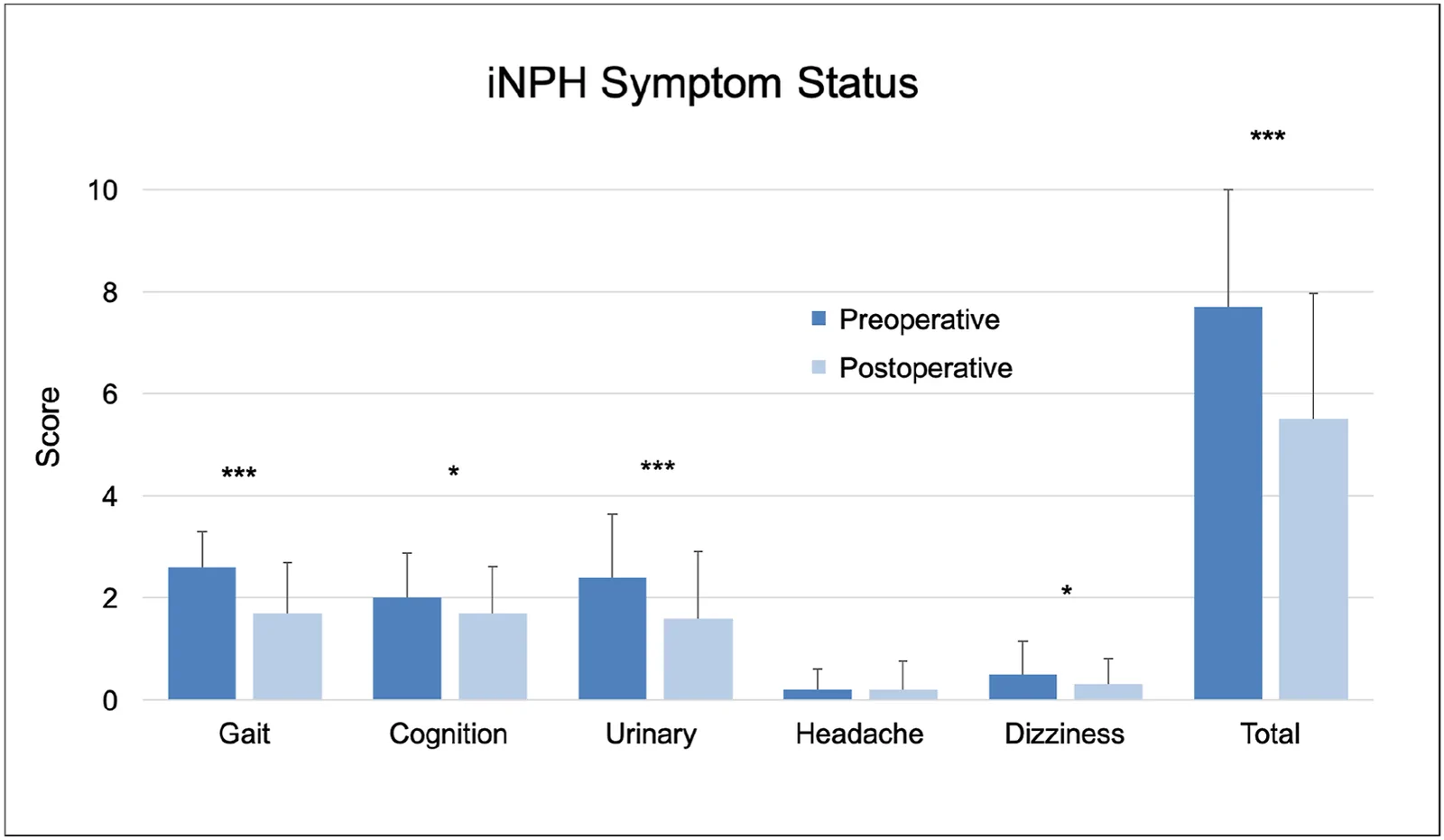
Symptom status of iNPH patients (n = 35) at *baseline (dark blue) and follow-up (light blue) as assessed* by ES score. Data are mean and SD, p values indicate statistically significant differences (*p < 0.05; ***p < 0.001).

The greatest improvement was observed for gait disturbance, with 74% patients improving by ≥ 1 points (p < 0.001). Urinary incontinence improved in 66% (p < 0.001), while cognitive impairment improved in 37% of patients (p = 0.03). Only a few patients experienced improvement in headaches (11%), whereas 29% of the patients reported reduced dizziness (p = 0.02) (Fig. 3). Detailed results for all categories are provided in Table 3. As described above, a second f/u was performed at a median of 283 days (range 152-426) including 80% of patients. At this time point, no consistent trend toward further improvement was observed compared to the initial f/u.

In multiple linear regression analyses, age, gender, CCI, and symptom duration were included as predictors of postoperative improvement (Δ iNPH symptom score). The overall model explained 12.6% of the variance (R^2^ = 0.13; adjusted R^2^ = 0.01), with no individual predictor reaching statistical significance. Age (β = −0.09, p = 0.077) and CCI (β = 0.55, p = 0.074) showed non-significant trends, whereas symptom duration and gender were not associated with postoperative improvement. Similar findings were observed in additional multiple linear regression models including extent of diagnostic work-up and educational level, with no significant associations identified.

### 3.4 Outcome expectation data

Outcome expectations differed between rater groups (Table 3). Mean OEQ score were 3.0 (SD 2.1) for PCG, 4.5 (SD 1.8) for residents, and 4.6 (SD 2.0) for neurosurgical specialists. The relation between the expected outcomes and observed outcomes is illustrated in the scatterplots (Fig. 4a, Fig. 4b, Fig. 4ca–c). A closer alignment with the observed ES score was seen for physician ratings compared to PCG, as reflected by tighter clustering of data points along the line of identity. This was supported by the Pearson correlation analysis, demonstrating the strongest association for neurosurgical specialists (r = 0.79), followed by residents (r = 0.74), whereas PCG ratings showed a lower, moderate correlation (r = 0.56).

**Fig. 4a:**
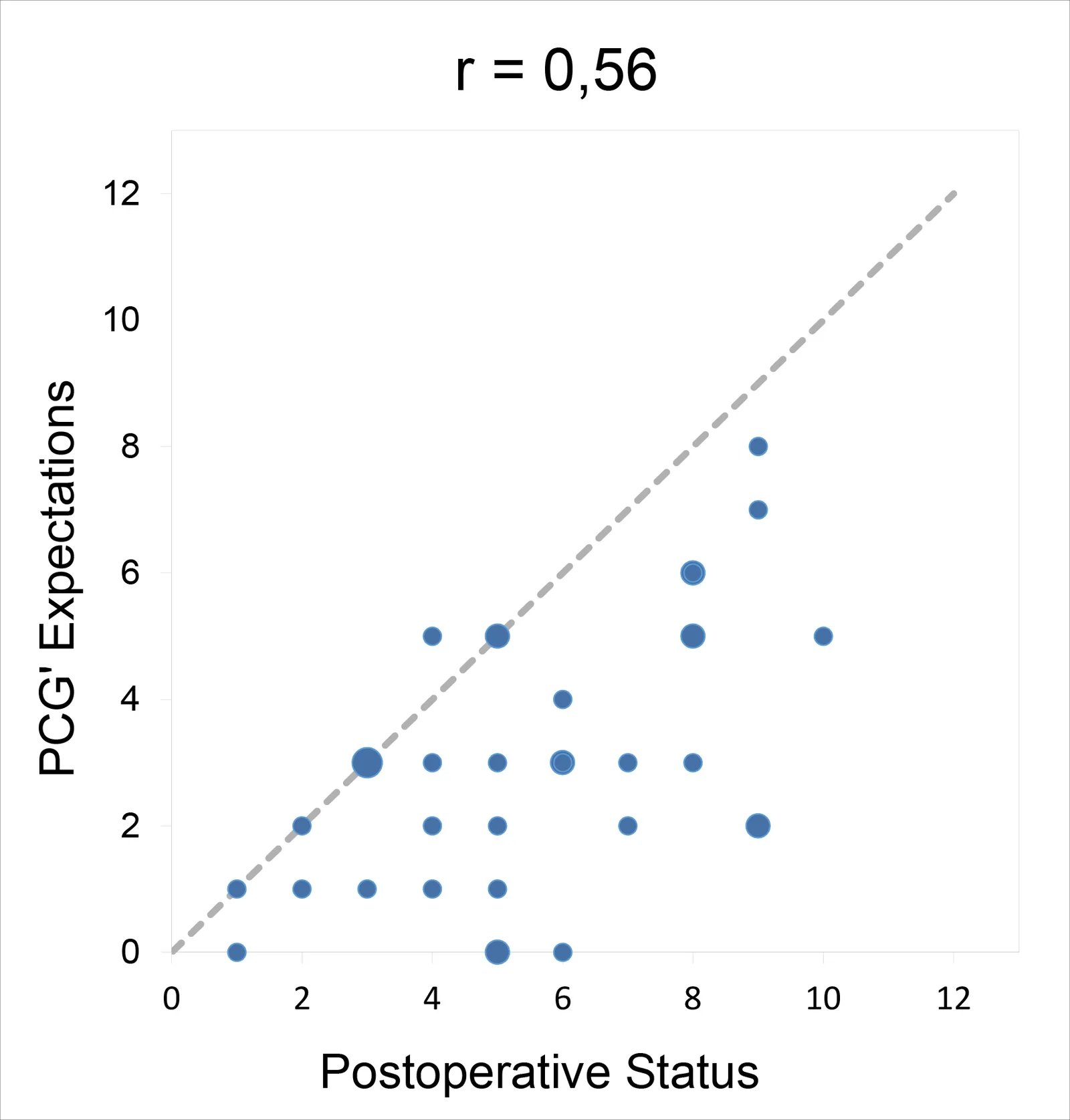
Scatterplot illustrating the Pearson correlation between the primary caregivers' (PCG) expectations and postoperative iNPH patient status. Larger dots indicate overlapping data points.

**Fig. 4b:**
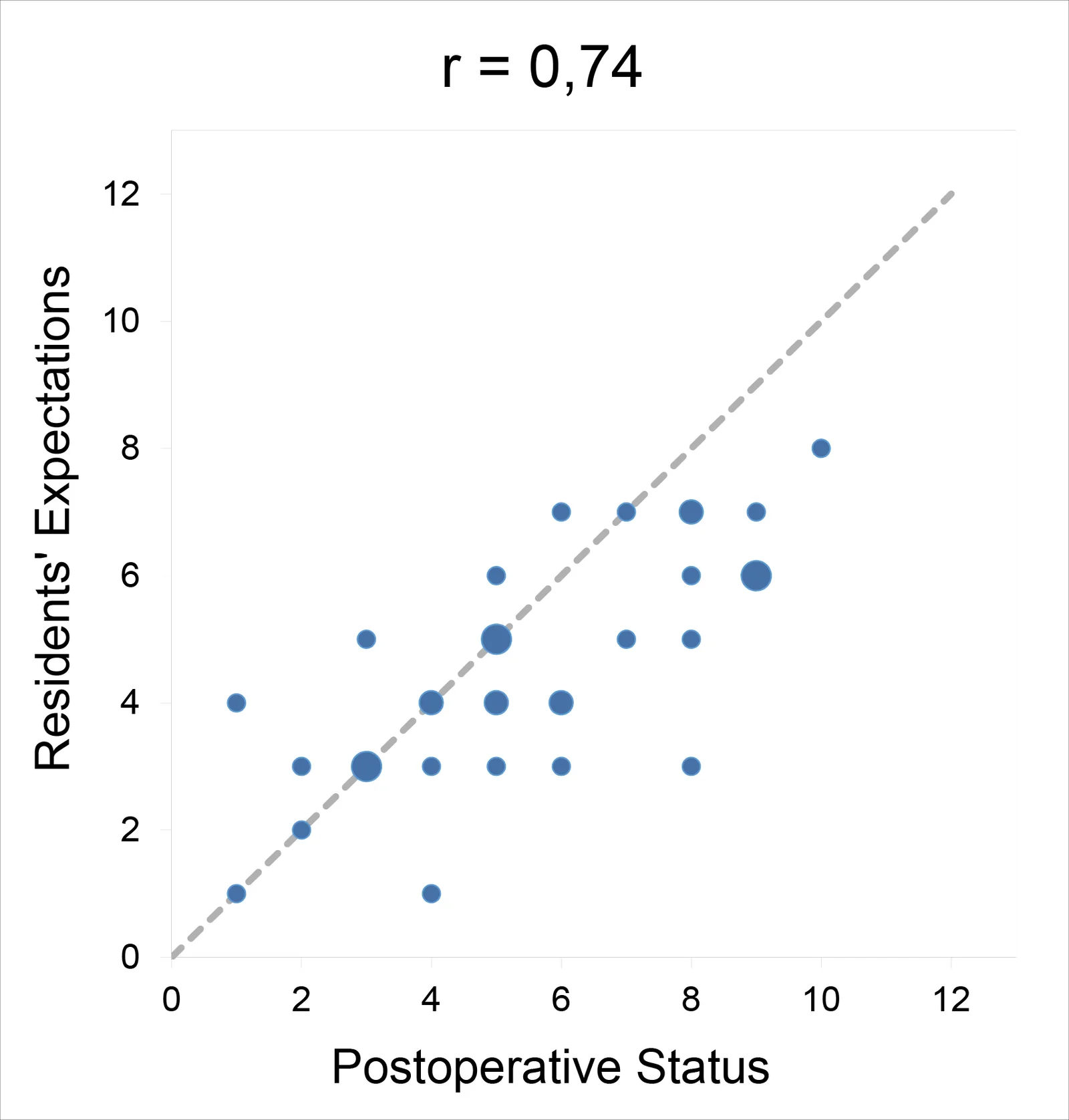
Scatterplot illustrating the Pearson correlation between the residents' expectations and postoperative iNPH patient status. Larger dots indicate overlapping data points.

**Fig. 4c:**
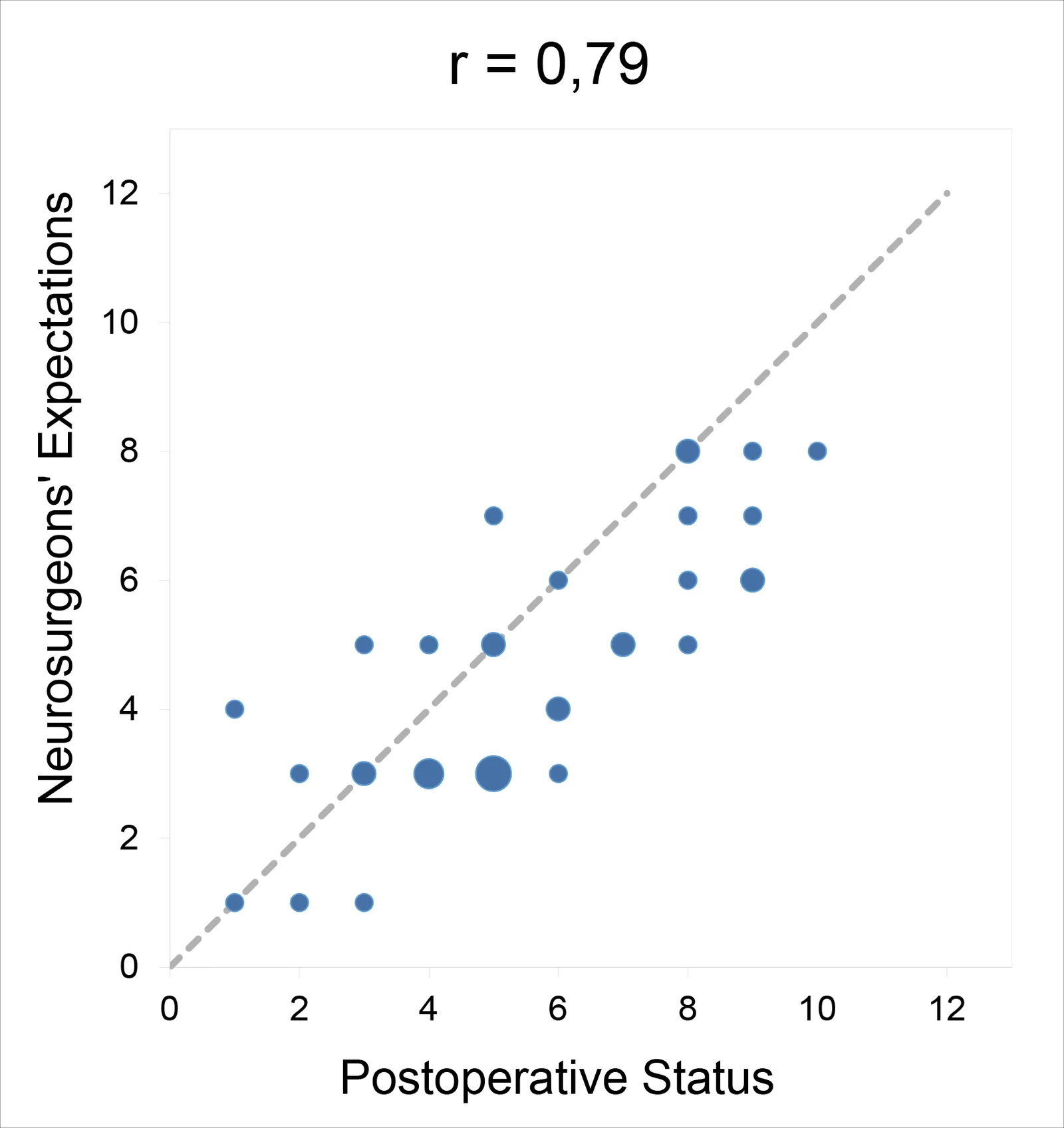
Scatterplot illustrating the Pearson correlation between the neurosurgeons' expectations and postoperative iNPH patient status. Larger dots indicate overlapping data points.

Linear mixed-effects models were used to account for repeated ratings within patients. Baseline iNPH scores and actual postoperative outcome were strongly associated with ratings (β = 0.61, p < 0.001 and β = −0.82, p < 0.001, respectively), and significant systematic differences between raters were observed (overall p < 0.001), indicating interrater variability. Age was also significantly associated with ratings (β = 0.07, p = 0.011), whereas gender, CCI, symptom duration, educational level, and extent of diagnostic work-up were not significantly associated with ratings. No significant interaction effects involving rater, educational level, or extent of diagnostic wok-up were observed. The patient-level ICC was 0.14, indicating modest clustering of ratings within patients.

## 4 Discussion

The primary aim of this study was to compare expected and observed treatment outcomes by relating postoperative symptom change to preoperative predictions from different observer groups. The findings demonstrate marked discordance between caregiver and medical professional expectations in a patient cohort representative of the disease. The presented data are consistent with the typical demographics, clinical presentation and outcome profile of iNPH.

### 4.1 Baseline characteristics

The mean age of 74.1 years and the male predominance are consistent with large observational cohorts, including a Swedish series of 3000 patients reporting a mean age of 74.4 years and a similar gender distribution (Sundström et al., 2022). The long duration of symptoms before diagnosis further illustrates the well-known diagnostic delay in iNPH, with most patients in our cohort symptomatic for more than one year before treatment. This is in line with published estimates showing that symptom duration often extends over 22 to 43 months before definitive diagnosis and intervention (Kazui et al., 2015; Agerskov et al., 2018; Pujari et al., 2008).

Comorbidity burden was substantial in our cohort, as reflected by a CCI of 3 or higher in 86% of patients. This is comparable to previous reports indicating that the vast majority of patients with iNPH present with at least one relevant comorbidity at the time of diagnosis (Luciano et al., 2025). Although the CCI captures overall disease burden and is an established predictor of survival, it has not been shown to predict functional outcome after shunt surgery (Klinge et al., 2023). Thus comorbidity burden in iNPH may influence symptom interpretation without necessarily determining treatment response.

Diagnosis in this cohort was based on established clinical and radiological criteria. In the majority of cases, these were supplemented by invasive testing, corresponding to probable iNPH according to current guidelines (Nakajima et al., 2021; Relkin et al., 2005). However, in selected cases invasive testing was omitted when the full Hakim triad was present. The necessity of invasive CSF diagnostics remains debated, as several studies suggest that patients may benefit from shunt treatment even if strict guideline criteria are not fully met (Wikkelso et al., 2013; Wu et al., 2020; Ishikawa et al., 2012; Tisell et al., 2011).

### 4.2 Outcome data

Follow-up was performed at a median of 48 days after shunt implantation. Although the f/u interval does not permit conclusions regarding long-term outcome, it was chosen deliberately to capture early therapeutic response. Previous studies have reported secondary deterioration within the first six months following noticeable treatment response (Gencer et al., 2024; Takeuchi and Yajima, 2019), although this is not supported by our second f/u data.

Postoperative improvement was seen in 83% of patients, with the mean iNPH symptom score decreased from 7.7 to 5.5, indicating a clear treatment effect. This is consistent with reported shunt response rates of approximately 71% to 93% in the literature (Wikkelso et al., 2013; Kazui et al., 2015; Pujari et al., 2008; Takeuchi and Yajima, 2019; Giordan et al., 2019). Consistent with previous reports, gait showed the strongest postoperative improvement. Luciano et al. reported improved walking speed in up to 80% of patients 3 months after treatment (Luciano et al., 2025). Kazui et al. assessed outcomes after lumboperitoneal shunt implantation using the Modified Ranking Scale and iNPHGS, and reported significant improvement in overall symptoms (mean Δ −2.2 points) as well as in gait disturbance (mean Δ −0.9 points) (Kazui et al., 2015). These findings closely mirror our results. In the SINPHONI-2 study, significant improvements in cognitive impairment and urinary incontinence were observed 3 months after CSF diversion surgery. Similarly, our cohort demonstrated significant improvement in urinary function, whereas cognitive improvement was less pronounced. This may be related to the shorter f/u interval, since previous studies suggest that cognitive recovery may continue during longer-term f/u (Caruso et al., 2024), potentially leading to an underestimation of cognitive benefit in our cohort. Headache and dizziness are not considered classic symptoms of iNPH but have previously been examined in the context of shunt treatment (Kiefer et al., 2003; Kuser et al., 2025). Dizziness improved in 29% of patients, whereas headache severity remained largely unchanged, likely because headaches more commonly reflect shunt overdrainage than iNPH itself (Sundström et al., 2022; Pujari et al., 2008; Kuser et al., 2025). The multiple linear regression analyses did not show a significant association between the extent of diagnostic work-up and postoperative improvement, nor did they identify a significant effect of CCI on outcome. Age and symptom duration were likewise not significantly associated with postoperative improvement. Given the relatively small sample size, these findings should be interpreted cautiously, as limited statistical power may have precluded detection of smaller effects.

Revision surgeries were required in 6 patients, corresponding to rates reported in previous studies. In a meta-analysis, 16% of patients required a second surgery when all types of CSF drainage implants were considered, and this increased to 18% when only VP shunts were considered (Giordan et al., 2019). In the same meta-analysis, 40% of patients exhibited symptoms of overdrainage, although most cases were managed by valve adjustments alone. Within our study cohort, shunt valve adjustments were necessary in 14% of cases.

### 4.3 Expectation data

The OEQ was introduced for this study, as no validated instrument is currently available to assess treatment outcome expectations in iNPH. Like the ES, it was based on established disease-specific grading scales, allowing observed and predicted outcomes to be compared using the same disease-specific dimension.

When OEQ ratings were compared with the actual postoperative outcome as assessed by the ES, neurosurgical specialists and residents showed the strongest agreement, whereas PCG were more optimistic. This pattern is in line with prior research showing that physicians generally predict surgical outcome more accurately than patients or relatives (Aoude et al., 2021; Witiw et al., 2018). According to Mancuso et al., estimates of treatment outcome two years after spinal surgery were more accurate among surgeons than among patients (Mancuso et al., 2021). Likewise, Lattig et al. demonstrated low Spearman correlations between physicians and patients (r_s_ = 0.097 to 0.222) when assessing the outcome after spinal surgery (Lattig et al., 2013). Findings from the Pearson correlation analyses were supported by the linear mixed model, indicating that ratings were systematically associated with baseline iNPH score and postoperative outcome measures. This suggests that treatment expectations reflected structured integration of clinical information. The strong correlation between predicted and observed outcome among neurosurgical raters suggests that disease-specific expertise may substantially improve prognostic judgment. Clear preoperative communication may therefore be particularly beneficial for patients and their relatives. Their expectations, individual concerns, treatment goals, and preferences should be explicitly elicited and discussed alongside the physician's disease-specific prognosis to facilitate realistic expectations and shared decision-making. Furthermore, counselling should provide clear and accessible information, avoiding technical jargon, and use visual resources where appropriate. While patient education in this study followed standard procedures, additional decision-support tools were not implemented. Previous studies have shown that decision aids improve patient knowledge and reduce decisional conflict during the surgical consent process, although effects on postoperative satisfaction appear limited (Knops et al., 2013; Pacheco-Brousseau et al., 2021; Stacey et al., 2024).

As not the patients themselves but their PCGs were evaluated, these findings also support the view that the perceived benefit of iNPH treatment extends beyond the patient. This interpretation is compatible with previous work demonstrating that caregiver strain decreased in parallel with treatment success (Kazui et al., 2011). These findings suggest that treatment effects in iNPH should be interpreted not solely on the basis of symptom reduction, but within a broader psychosocial context that also includes outcomes related to caregivers.

### 4.4 Limitations

This study has several limitations that should be considered when interpreting the results. First, the assessment of expectations was based on questionnaire data and is therefore inherently susceptible to bias. In addition, the iNPH symptom scoring was originally developed to assess clinical outcome, and the OEQ itself has not been validated for assessing treatment expectations. Second, the relatively small cohort of 35 patients may have reduced the statistical power to detect smaller associations and precluded robust subgroup analyses. The single-centre design may further limit the generalisability of the findings to other patient populations and clinical settings. Ultimately, the short follow-up interval allowed assessment of early postoperative response only and does not permit conclusions regarding long-term treatment outcome.

## 5 Conclusion

In iNPH, postoperative improvement is common, but expectations are shaped differently depending on the observer's perspective. Neurosurgical expertise appears to align more closely with actual outcome than expectations held by primary caregivers. This suggests that structured preoperative counselling may help reduce mismatches between anticipated and observed benefit.

Future research should focus on whether targeted communication strategies, decision aids, or standardized counselling tools can improve expectation calibration before surgery. Such approaches may be especially valuable in iNPH, where symptom change is multidimensional and the perception of benefit may differ across patients, families, and clinicians.

## Ethics declaration

Written informed consent to take part in the study and to publish the article has been obtained from all participants or their legal representatives. The privacy rights of participants have been observed.

This study was performed in compliance with relevant laws, regulatory frameworks and guidelines where the research took place. This study was approved by the Ethikkomission an der TUM. (Approval No. 649/21 S)

The results of this clinical trial and any associated work have been posted in a registry. This clinical trial was registered with number ID: DRKS000310 (German Federal Institute for Drug and Medical Devices).

## Funding

This research did not receive any specific grant from funding agencies in the public, commercial, or not-for-profit sectors.

During the preparation of this work, the authors used Paperpal for language editing and grammatical revision. After using this tool, the authors reviewed and edited the content as needed and take full responsibility for the content of the published article.

## Declaration of competing interests

The authors declare that they have no known competing financial interests or personal relationships that could have appeared to influence the work reported in this paper.

## Glossary

CCI: Charlson Comorbidity Index
95% CI: 95% Confidence Intervals
CSF: Cerebrospinal Fluid
e.g.: For example
ES: Evaluation sheet
f/u: Follow-up
iNPH: Idiopathic Normal Pressure Hydrocephalus
MoCA: Montreal Cognitive Assessment
mRS: Modified Rankin Scale
NPH: Normal Pressure Hydrocephalus
OEQ: Outcome expectation questionnaire
PCG: Primary caregiver
SD: Standard deviation
VP: Ventriculoperitoneal

## References

[bib1] Agerskov S., Hellström P., Andrén K., Kollén L., Wikkelsö C., Tullberg M. (2018). The phenotype of idiopathic normal pressure hydrocephalus-a single center study of 429 patients. J. Neurol. Sci..

[bib2] Andersson J., Rosell M., Kockum K., Lilja-Lund O., Söderström L., Laurell K., Burger M.C. (2019). Prevalence of idiopathic normal pressure hydrocephalus: a prospective, population-based study. PLoS One.

[bib3] Aoude A., Litowski M., Aldebeyan S., Fisher C., Hall H., Manson N. (2021). A comparison of patient and surgeon expectations of spine surgical outcomes. Glob. Spine J..

[bib4] Caruso J.P., El Ahmadieh T.Y., Trent T., Stutzman S.E., Anderson R., Schneider N. (2024). Neurologic quality of life outcomes in patients with normal pressure hydrocephalus after Ventriculoperitoneal shunt placement: a prospective assessment of cognition, mobility, and social participation. World Neurosurg..

[bib5] Gencer A.H., Schwarm F.P., Nagl J., Uhl E., Kolodziej M.A. (2024). The benefits of ventriculoperitoneal shunting in normal pressure hydrocephalus patients—a follow-up of three years. Acta Neurochir..

[bib6] Giordan E., Palandri G., Lanzino G., Murad M.H., Elder B.D. (2019). Outcomes and complications of different surgical treatments for idiopathic normal pressure hydrocephalus: a systematic review and meta-analysis. J. Neurosurg..

[bib7] Ishikawa M., Hashimoto M., Mori E., Kuwana N., Kazui H. (2012). The value of the cerebrospinal fluid tap test for predicting shunt effectiveness in idiopathic normal pressure hydrocephalus. Fluids Barriers CNS.

[bib8] Jaraj D., Rabiei K., Marlow T., Jensen C., Skoog I., Wikkelsø C. (2014). Prevalence of idiopathic normal-pressure hydrocephalus. Neurology.

[bib10] Kazui H., Miyajima M., Mori E., Ishikawa M. (2015). Lumboperitoneal shunt surgery for idiopathic normal pressure hydrocephalus (SINPHONI-2): an open-label randomised trial. Lancet Neurol..

[bib9] Kazui H., Mori E., Hashimoto M., Ishikawa M., Hirono N., Takeda M. (2011). Effect of shunt operation on idiopathic normal pressure hydrocephalus patients in reducing caregiver burden: evidence from SINPHONI. Dement. Geriatr. Cogn. Disord.

[bib11] Kiefer M., Eymann R., Komenda Y., Steudel W.I. (2003). [A grading system for chronic hydrocephalus]. Zentralbl. Neurochir..

[bib12] Klinge P.M., Ma K.L., Leary O.P., Sastry R.A., Sayied S., Venegas O. (2023). Charlson comorbidity index applied to shunted idiopathic normal pressure hydrocephalus. Sci. Rep..

[bib13] Knops A.M., Legemate D.A., Goossens A., Bossuyt P.M.M., Ubbink D.T. (2013). Decision aids for patients facing a surgical treatment decision: a systematic review and meta-analysis. Ann. Surg..

[bib14] Kubo Y., Kazui H., Yoshida T., Kito Y., Kimura N., Tokunaga H. (2008). Validation of grading scale for evaluating symptoms of idiopathic normal-pressure hydrocephalus. Dement. Geriatr. Cogn. Disord.

[bib15] Kuser F., Dias S.F., Stieglitz L.H. (2025). Long-Term Follow-up in idiopathic normal pressure Hydrocephalus—Towards a more comprehensive disease outcome. World Neurosurg..

[bib16] Lattig F., Fekete T.F., O'Riordan D., Kleinstuck F.S., Jeszenszky D., Porchet F. (2013). A comparison of patient and surgeon preoperative expectations of spinal surgery. Spine Phila Pa.

[bib17] Luciano M.G., Williams M.A., Hamilton M.G., Katzen H.L., Dasher N.A., Moghekar A. (2025). A randomized trial of shunting for idiopathic normal-pressure hydrocephalus. N. Engl. J. Med..

[bib18] Mancuso C.A., Duculan R., Cammisa F.P., Sama A.A., Hughes A.P., Lebl D.R. (2021). Concordance between Patients' and Surgeons' expectations of lumbar surgery. Spine.

[bib19] Martín-Láez R., Caballero-Arzapalo H., López-Menéndez L.Á., Arango-Lasprilla J.C., Vázquez-Barquero A. (2015). Epidemiology of idiopathic normal pressure hydrocephalus: a systematic review of the literature. World Neurosurg..

[bib20] Nakajima M., Yamada S., Miyajima M., Ishii K., Kuriyama N., Kazui H. (2021). Guidelines for management of idiopathic normal pressure hydrocephalus (Third edition). Endorsed by the Japanese Society of Normal Pressure Hydrocephalus. Neurol Med Chir (Tokyo).

[bib21] Pacheco-Brousseau L., Charette M., Poitras S., Stacey D. (2021). Effectiveness of patient decision aids for total hip and knee arthroplasty decision-making: a systematic review. Osteoarthr. Cartil..

[bib22] Pearce R.K.B., Gontsarova A., Richardson D., Methley A.M., Watt H.C., Tsang K. (2024). Shunting for idiopathic normal pressure hydrocephalus. Cochrane Z_INACTIVE_Dementia and Cognitive Improvement Group. Cochrane Database Syst. Rev..

[bib23] Pujari S., Kharkar S., Metellus P., Shuck J., Williams M.A., Rigamonti D. (2008). Normal pressure hydrocephalus: long-term outcome after shunt surgery. J. Neurol. Neurosurg. Psychiatry.

[bib24] Relkin N., Marmarou A., Klinge P., Bergsneider M., Black PMcL. (2005). Diagnosing idiopathic normal-pressure hydrocephalus. Neurosurgery.

[bib25] Rönnberg K., Lind B., Zoëga B., Halldin K., Gellerstedt M., Brisby H. (2007). Patients' satisfaction with provided care/information and expectations on clinical outcome after lumbar disc herniation surgery. Spine.

[bib26] Schniepp R., Trabold R., Romagna A., Akrami F., Hesselbarth K., Wuehr M. (2017). Walking assessment after lumbar puncture in normal-pressure hydrocephalus: a delayed improvement over 3 days. J. Neurosurg..

[bib27] Soroceanu A., Ching A., Abdu W., McGuire K. (2012). Relationship between preoperative expectations, satisfaction, and functional outcomes in patients undergoing lumbar and cervical spine surgery: a multicenter Study. Spine.

[bib28] Stacey D., Lewis K.B., Smith M., Carley M., Volk R., Douglas E.E. (2024). Decision aids for people facing health treatment or screening decisions. Cochrane Consumers and Communication Group. Cochrane Database Syst. Rev..

[bib29] Subramanian H.E., Mahajan A., Sommaruga S., Falcone G.J., Kahle K.T., Matouk C.C. (2018). The subjective experience of patients undergoing shunt surgery for idiopathic normal pressure hydrocephalus. World Neurosurg..

[bib30] Sundström N., Lundin F., Arvidsson L., Tullberg M., Wikkelsø C. (2022). The demography of idiopathic normal pressure hydrocephalus: data on 3000 consecutive, surgically treated patients and a systematic review of the literature. J. Neurosurg..

[bib31] Takeuchi T., Yajima K. (2019). Long-term 4 years Follow-up Study of 482 patients who underwent shunting for idiopathic normal pressure hydrocephalus -Course of symptoms and shunt efficacy rates compared by Age Group-. Neurol. Med.-Chir..

[bib32] Tisell M., Tullberg M., Hellström P., Edsbagge M., Högfeldt M., Wikkelsö C. (2011). Shunt surgery in patients with hydrocephalus and white matter changes: clinical article. J. Neurosurg..

[bib33] Toma A.K., Tarnaris A., Kitchen N.D., Watkins L.D. (2011). Working towards patient oriented outcome assessment in normal pressure hydrocephalus, what is the most important?. Acta Neurochir..

[bib34] von Elm E., Altman D.G., Egger M., Pocock S.J., Gøtzsche P.C., Vandenbroucke J.P. (2007). The strengthening the reporting of Observational Studies in Epidemiology (STROBE) statement: guidelines for reporting observational studies. Lancet.

[bib35] Wikkelso C., Hellstrom P., Klinge P.M., Tans J.T.J. (2013). On behalf of the European iNPH Multicentre Study Group. The European iNPH Multicentre Study on the predictive values of resistance to CSF outflow and the CSF Tap Test in patients with idiopathic normal pressure hydrocephalus. J. Neurol. Neurosurg. Psychiatry.

[bib36] Witiw C.D., Mansouri A., Mathieu F., Nassiri F., Badhiwala J.H., Fessler R.G. (2018). Exploring the expectation-actuality discrepancy: a systematic review of the impact of preoperative expectations on satisfaction and patient reported outcomes in spinal surgery. Neurosurg. Rev..

[bib37] Wu E.M., El Ahmadieh T.Y., Kafka B., Caruso J.P., Neeley O.J., Plitt A.R. (2020). Clinical outcomes of normal pressure hydrocephalus in 116 patients: objective versus subjective assessment. J. Neurosurg..

