# Supplementary material for: Expectations of cerebrospinal fluid shunt treatment outcome in idiopathic normal pressure hydrocephalus: a combined cross-sectional and prospective cohort study
Source: Brain Spine. 2026 Sep 5;6:106664. doi: 10.1016/j.bas.2026.106664 (PMC13578621; doi:10.1016/j.bas.2026.106664)
Supplement: Supplementary file 1 [file mmc1.docx]

Clinical “Evaluation Sheet” – Hydrocephalus Status

Questionnaire:

Expectations of Hydrocephalus Treatment: Comparison between Caregiver and Surgeon

Evaluation sheet for patient (please enter initials):

Evaluation sheet completed (please enter date):

Evaluation sheet completed (please tick)

Preoperative	(	)

Postoperative	(	)

1) **Current gait status:**
   - Normal gait
   - Mild/minimal gait disturbance
     Veering, staggering or wobbling while walking
   - Moderate gait disturbance	Unstable but independent gait
   - Severe gait disturbance	Walking with support only (walking with a walking aid or	assistance from another person)
   - Walking not possible
2) **Current cognitive status:**
   - Normal cognitive function, with no cognitive impairment
   - Mild/minimal cognitive impairment	Minimal complaints of amnesia or inattention
   - Moderate cognitive impairment	Marked complaints of amnesia or inattention, but no	disorientation
   - Severe cognitive impairment
     Disorientation to time (day of the week, date) and place (current	city, place of residence), but conversation is possible
   - Profound cognitive impairment
     Disorientation for the situation or meaningful conversation	impossible
3) **Current urinary status:**
   - Normal urinary function
   - Mild urinary impairment
     Urinary urgency cannot be suppressed for long, pollakiuria
   - Moderate urinary impairment
     Occasional urinary incontinence (several times per week, but not	daily)
   - Severe urinary impairment
     Continuous urinary incontinence (once or more per day)
   - Complete urinary incontinence
     No control of bladder emptying
4) **Current headache status:**
   - No headaches
   - Intermittent or permanent headache which does not influence the patient’s activities of daily life
   - Persistent severe headache
5) **Current dizziness status:**
   - No dizziness
   - Intermittent dizziness occurring during exertion or spontaneous dizziness
   - Persistent dizziness
