# Supplementary material for: Expectations of cerebrospinal fluid shunt treatment outcome in idiopathic normal pressure hydrocephalus: a combined cross-sectional and prospective cohort study
Source: Brain Spine. 2026 Sep 5;6:106664. doi: 10.1016/j.bas.2026.106664 (PMC13578621; doi:10.1016/j.bas.2026.106664)
Supplement: Supplementary file 3 [file mmc3.docx]

Physician Questionnaire – Hydrocephalus Status

Questionnaire:

Expectations of Hydrocephalus Treatment: Comparison between Caregiver and Surgeon

Questionnaire for patient (please enter initials):

Questionnaire completed (please enter date):

Questionnaire completed by (please tick)

Neurosurgical specialist	(	)

Resident	(	)

Dear colleague,

Please complete this questionnaire after independently examining the patient and independently reviewing the relevant previous findings and investigations. We aim to assess how effectively we inform patients and their relatives about hydrocephalus and the planned treatment. To investigate expectations regarding the subsequent operation from both the medical and patient perspectives, the responses to this questionnaire will be evaluated. Please try to imagine what treatment outcome you expect the patient to achieve approximately 6 weeks after the operation, for example with regard to walking ability, cognitive function, or expected bladder function. Please try to consider realistic treatment outcomes. We are interested in your expectations regarding the treatment outcome; we are not asking about the outcome you hope for.

Thank you very much for your participation.

How do you generally assess the potential treatment outcome, and how do you expect the patient’s symptoms to change as a result of the treatment?

1) I expect the patient to have the following walking ability 6 weeks after treatment:
   - Normal gait
   - Mild/minimal gait disturbance
     Veering, staggering or wobbling while walking
   - Moderate gait disturbance	Unstable but independent gait
   - Severe gait disturbance	Walking with support only (walking with a walking aid or	assistance from another person)
   - Walking not possible
2) I expect the patient to have the following cognitive function 6 weeks after treatment:
   - Normal cognitive function, with no cognitive impairment
   - Mild/minimal cognitive impairment	Minimal complaints of amnesia or inattention
   - Moderate cognitive impairment	Marked complaints of amnesia or inattention, but no	disorientation
   - Severe cognitive impairment
     Disorientation to time (day of the week, date) and place (current	city, place of residence), but conversation is possible
   - Profound cognitive impairment
     Disorientation for the situation or meaningful conversation	impossible
3) I expect the patient to have the following degree of bladder control 6 weeks after treatment:
   - Normal urinary function
   - Mild urinary impairment
     Urinary urgency cannot be suppressed for long, pollakiuria
   - Moderate urinary impairment
     Occasional urinary incontinence (several times per week, but not	daily)
   - Severe urinary impairment
     Continuous urinary incontinence (once or more per day)
   - Complete urinary incontinence
     No control of bladder emptying
4) I expect the patient to have the following headache symptoms 6 weeks after treatment:
   - No headaches
   - Intermittent or permanent headache which does not influence the patient’s activities of daily life
   - Persistent severe headache
5) I expect the patient to have the following dizziness symptoms 6 weeks after treatment:
   - No dizziness
   - Intermittent dizziness occurring during exertion or spontaneous dizziness
   - Persistent dizziness
